# Measuring polio immunity to plan immunization activities

**DOI:** 10.1016/j.vaccine.2016.10.017

**Published:** 2016-11-21

**Authors:** Arend Voorman, Hil M. Lyons

**Affiliations:** a The Bill and Melinda Gates Foundation, 500th 5th Ave N, Seattle, WA 98109, USA; b The Institute for Disease Modeling, 3150 139th Ave SE, Bellevue, WA 98005, USA

**Keywords:** Polio, Vaccination, Immunity, Surrogate marker, Data quality

## Abstract

The Global Polio Eradication Initiative is closer than ever to achieving a polio-free world. Immunization activities must still be carried out in non-endemic countries to maintain population immunity at levels which will stop poliovirus from spreading if it is re-introduced from still-infected areas. In areas where there is no active transmission of poliovirus, programs must rely on surrogate indicators of population immunity to determine the appropriate immunization activities, typically caregiver-reported vaccination history obtained from non-polio acute flaccid paralysis patients identified through polio surveillance. We used regression models to examine the relationship between polio vaccination campaigns and caregiver-reported polio vaccination history. We find that in many countries, vaccination campaigns have a surprisingly weak impact on these commonly used indicators. We conclude that alternative criteria and data, such as routine immunization indicators from vaccination records or household surveys, should be considered for planning polio vaccination campaigns, and that validation of such surrogate indicators is necessary if they are to be used as the basis for program planning and risk assessment. We recommend that the GPEI and similar organizations consider or continue devoting additional resources to rigorously study population immunity and campaign effectiveness in at-risk countries.

## Highlights

• Reliability and comparability of reported polio vaccine dose histories are studied.
• In many areas, reported doses have a weak relationship to immunization activities.
• Data quality may distort risk assessment and immunization activity allocation.
• Alternative data should be considered for planning polio immunization activities.

## 1 Introduction

Since the Global Polio Eradication Initiative (GPEI) began in 1988, the estimated number of cases of poliomyelitis has dropped by more than 99% [1]. As a result, the number of countries with circulation of wild poliovirus has decreased from over 120 to 3: Afghanistan, Pakistan, and Nigeria, with wild poliovirus discovered in Nigeria in August 2016 after a long period of silent circulation [2]. This dramatic progress has often been interrupted by re-importation of wild poliovirus into non-endemic countries. Since 2001 there have been wild polio outbreaks from re-introduction of virus into 41 previously polio-free countries [3], in addition to outbreaks of circulating vaccine-derived poliovirus in 22 countries [4]. Such outbreaks highlight the need to maintain high levels of population immunity globally as long as live poliovirus exists.

The GPEI relies on Supplementary Immunization Activities (SIAs) to improve areas with low population immunity. These SIAs, which provide polio vaccine through fixed posts and house-to-house vaccination campaigns, account for approximately 40% of the GPEI’s estimated 2013–2018 budget [5]. In 2013, SIAs reached over 250 million children in over 43 countries at a cost of approximately $607 million USD [5]. The frequency and geographic scope of SIAs varies widely depending on the perceived outbreak risk and perceived impact of SIAs on population immunity, from zero SIAs in areas with high routine immunization coverage, to over 9 SIAs per-year in polio-endemic countries [5]. Given their financial and epidemiological importance, judicious allocation of SIAs is a central task of the GPEI.

### 1.1 Assessing population immunity and polio outbreak risk

When exposure to wild poliovirus was widespread, the occurrence of polio cases identified populations with low immunity and in turn guided the GPEI in the number and geographic scope of SIAs [6]. However, in the absence of poliovirus detection, the GPEI must rely on surrogates of polio immunity and perceived risk of exposure to wild poliovirus to guide vaccination efforts.

The number of SIAs required to prevent transmission of poliovirus varies widely, depending on routine immunization levels, vaccination campaign coverage, vaccine efficacy, and local characteristics of the population that influence the transmissibility of poliovirus, such as population density and sanitation [7]. For an individual, the number of polio vaccine doses required for protection from polio varies with vaccine type, vaccine schedule, and public health factors including sanitation and prevalence of enteric pathogens [8]. For Type 1 poliovirus, estimates of protective efficacy from three doses of trivalent oral polio vaccine (tOPV) vary between 30 and 100% [8], [9], [10], [11]. Further, the fraction of immune individuals in the population required to stop transmission of poliovirus depends on seasonality, geographic context, and the type of poliovirus [12]. Thus, the number of doses that need to be delivered through a combination of routine immunization and SIAs varies. While precise vaccination targets are not used, for purposes of risk assessment countries are often considered low-risk if at least 90% of the population has three or more doses of OPV [3], in line with the broader immunization coverage targets advocated by the World Health Assembly [13].

For risk analysis, the GPEI relies heavily on immunization coverage estimates derived from reported polio vaccination history of non-polio acute flaccid paralysis (NP-AFP) cases [3], [14]. NP-AFP data is used for global risk assessment because it is comprehensively collected and maintained by the World Health Organization (WHO), does not rely on population estimates, and because of its presumed comparability across geographies. However, it is widely acknowledged that these data have several key weaknesses when used for risk analysis, including a relatively small sample size, a potentially non-representative sample of individuals, and incomplete record and recall of dose history.

Routine immunization indicators are also often used in risk assessment, typically based on household surveys or the WHO/United Nations Children’s Fund Estimates of National Immunization Coverage (WUENIC) [3], [14]. However, such indicators do not measure vaccination through SIAs, and may under-estimate polio immunity levels.

### 1.2 Assessing vaccination history

The polio vaccination history used in risk assessment is recorded on case investigation forms by local polio surveillance officers, typically from a parent or caregiver of the paralyzed child. When reporting vaccination history, the caregiver must consider vaccinations from two distinct sources: routine immunization and SIAs. Routine immunization typically occurs in a health clinic, and can be verified through vaccination cards when available, while vaccination through SIAs typically occurs at the child’s home and is not verifiable. Thus, in ascertaining polio vaccination history, polio surveillance officers must rely heavily on parental recall, which is less reliable and more sensitive to the survey instrument used [15].

Case investigation forms differ by country, though global and regional reporting requirements stipulate the minimum amount of information that is collected [16]. In countries in the African Region (AFR) of the WHO, the case investigation form asks for the total number of polio vaccine doses, as well as up to 5 vaccination dates. Notably, the case investigation forms in AFR do not distinguish between doses given by SIA and by the routine program. However, in countries in the Eastern Mediterranean Region (EMR) and the South East Asia Region (SEAR), case investigation forms must separately record doses received from routine immunization and SIAs. Examples of these survey instruments are available in the supplementary material.

In this paper, we examine how vaccination histories are influenced by SIAs, how this relationship varies between countries and regions, and how this affects risk assessment.

## 2 Data and methods

Acute flaccid paralysis (AFP) cases in children under 15 years of age are identified through the global polio surveillance network [17]. Once a case is identified, an investigation is initiated in which a surveillance officer collects basic clinical and demographic information, including polio vaccination history, on a case investigation form. Two stool samples are collected within 14 days of onset and within 24–48 h of each other and sent to a laboratory in the Global Polio Laboratory Network for testing. Clinical information and stool culture results are used to classify AFP cases as either polio or non-polio AFP. Regional and global databases of AFP case data are maintained by the WHO. From January 2010 to July 2015, 484,497 NP-AFP cases were identified in 75 countries.

The WHO also maintains a database of all supplemental polio immunization activities. The database includes information on the dates and location of the activity, the type of activity (National Immunization Day, Child Health Day, etc.), the target population, and the type of vaccine used. Campaign data from January 2000 to July 2015 were used.

**Fig. 1:**
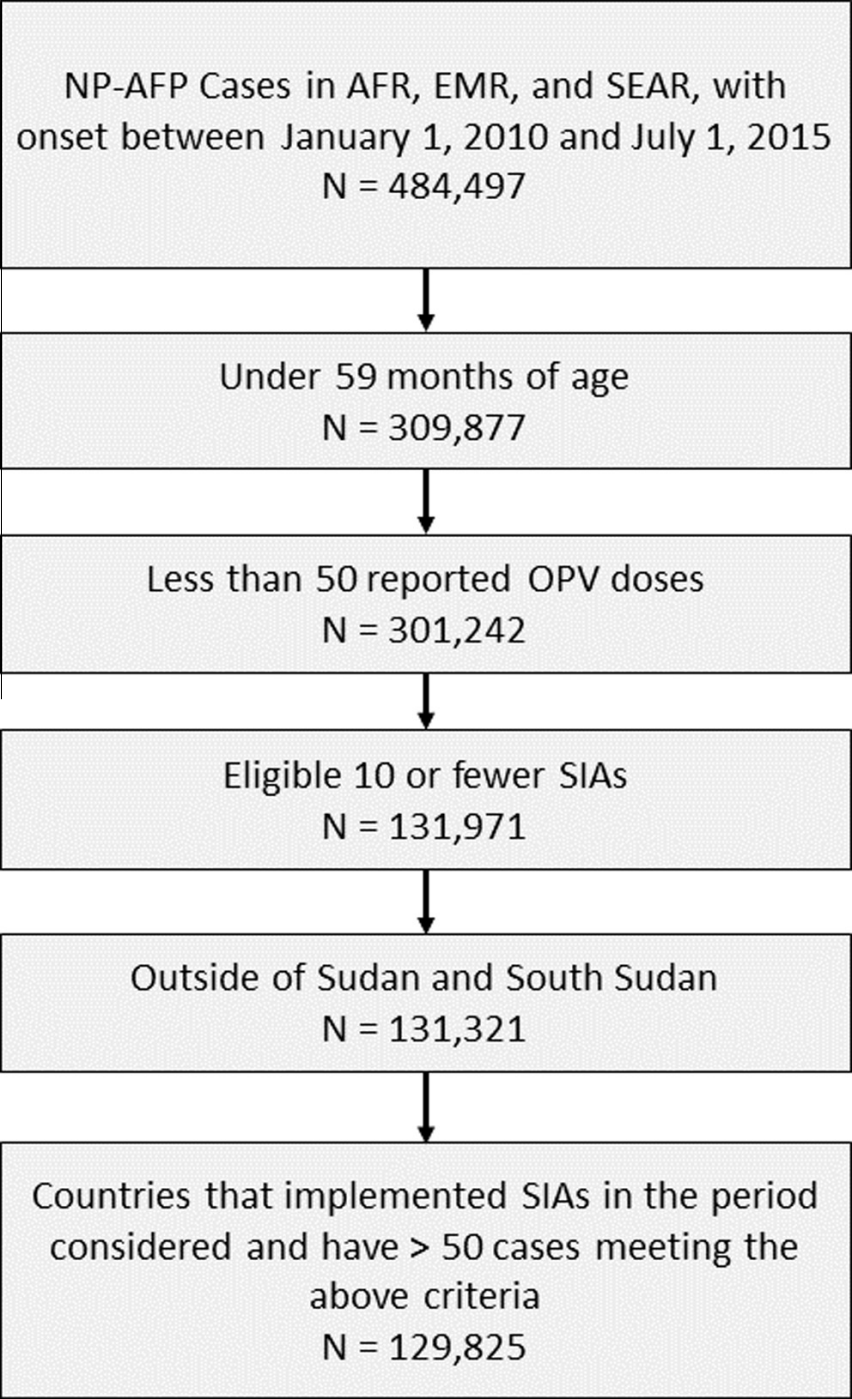
Schematic representation of exclusion criteria applied to the AFP database.

We matched the campaign and AFP databases to determine the number of vaccination campaigns occurring in each case’s district for which the child would be eligible, prior to the onset of paralysis. We excluded cases if they were over 59 months of age at the onset of paralysis, had experienced more than 10 SIAs, or reported more than 50 doses of OPV. These exclusions were applied since we do not expect accurate vaccination histories from older children who have experienced many SIAs, regardless of the method of ascertainment. In the Supplementary Material we examine the sensitivity of our analyses to this exclusion. We also restricted our analysis to countries with 50 or more NP-AFP cases not excluded from the above criteria, and that had conducted more than one SIA over the time period considered. We omitted data from South Sudan and Sudan, where recent administrative changes complicate identification of campaign history. This resulted in 129,825 cases in 47 countries used in the primary analysis (see Fig. 1). Table 1 summarizes characteristics of the children included in the analysis, by region. Summary tables of all children by country are in the Supplementary Material.

For our primary analysis, we characterized the relationship between caregiver-reported doses from NP-AFP cases and the number of vaccination campaigns experienced by the case using a linear regression model, adjusted for province to account for differences in routine immunization coverage. This model takes the form

$$\mathrm{Model}\;1:\;{\mathrm{Doses}}_{\mathrm{ij}}=\alpha_{\mathrm{Aj}}+\beta_{A}\times (\#\mathrm{SIAs}\;\mathrm{experienced})+{∊}_{i}\text{,}$$

where ${\mathrm{Doses}}_{\mathrm{ij}}$ are the doses reported by person i in province j, $\alpha_{\mathrm{Aj}}$ is the average number of doses reported in the absence of campaigns, $\beta_{A}$ is the increase in average doses associated with each additional campaign experienced, and ${∊}_{i}$ is a mean-zero error term. This model estimates the increase in the expected number of doses reported on a case investigation form for each additional vaccination campaign experienced, averaged across provinces over the years 2010–2015. The model is fit separately for each country. When the reported number of doses is unbiased $\beta_{A}$ is an estimate of average campaign coverage. However, due to imperfect ascertainment of dose histories, the estimate is more correctly interpreted as the marginal impact of a vaccination campaign on the average number of doses reported on a case investigation form.

We also examined the relationship between the number of SIAs experienced and the proportion of children reporting zero OPV doses (the zero-dose fraction), as well as the proportion of children reporting less than three OPV doses (also termed the under-immunized fraction), since these are the traditional indicators used in risk assessment. These models take the form:

$$\mathrm{Model}\;2:\;\mathrm{Pr}({\mathrm{Doses}}_{\mathrm{ij}}<3)=\alpha_{\mathrm{uj}}\times {\left(1-\beta_{U}\right)}^{\#\mathrm{SIAs}\;\mathrm{experienced}}\text{,}\;\text{and}$$

$$\mathrm{Model}\;3:\;\mathrm{Pr}({\mathrm{Doses}}_{\mathrm{ij}}=0)=\alpha_{\mathrm{zj}}\times {\left(1-\beta_{Z}\right)}^{\#\mathrm{SIAs}\;\mathrm{experienced}}\text{.}$$

Here, $\alpha_{\mathrm{uj}}$ and $\alpha_{\mathrm{zj}}$ are the under-immunized and zero-dose fractions, respectively, in the absence of vaccination campaigns. Likewise, $\beta_{Z}$ and $\beta_{u}$ are the proportional decrease in zero-dose fraction and under-immunized fraction, respectively, associated with each additional SIA. Models 2 and 3 are fit using modified Poisson regression for binary data [18]. In Models 2 and 3 we omitted countries with 10 or fewer under-immunized or zero-dose cases.

Our primary interest was in characterizing the plausible impact of SIAs on reported doses, using point-estimates and 95% confidence intervals. We also note where confidence intervals include 0, corresponding to a 2-sided hypothesis test of $H_{0}:\beta =0$ and a Type 1 Error rate of 0.05 per-test. In all analyses, we used model-robust standard errors which permit valid inference on trends even when the model is not correct [19], [20].

## 3 Results

### 3.1 Relationship between reported doses and vaccination campaigns experienced

**Fig. 2:**
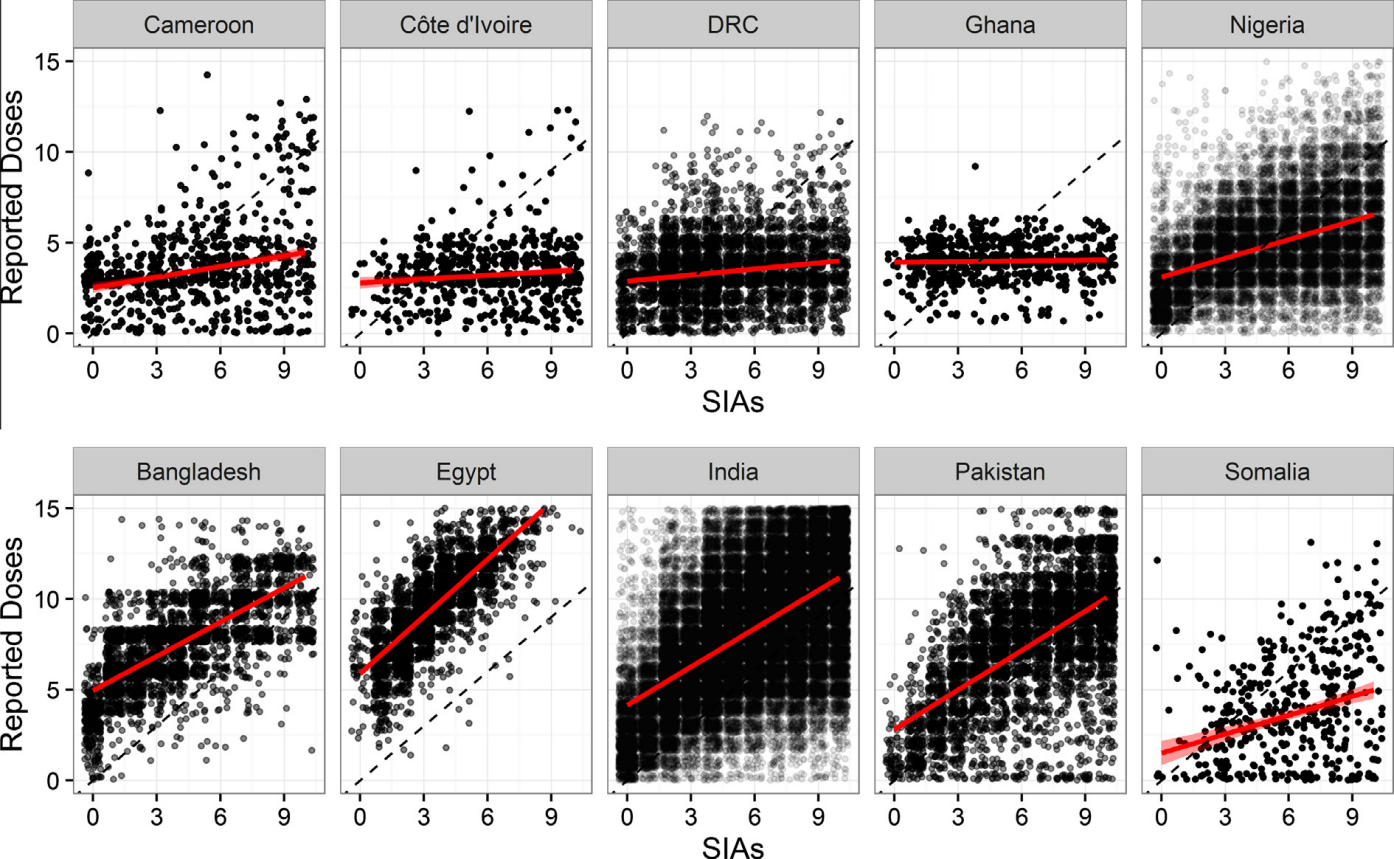
Total number of OPV doses reported and the number of vaccination campaigns experienced by a child, based on NP-AFP data between January 2010 and July 2015. Red lines indicate simple linear regression fit and 95% confidence interval. Countries in top panel are in the AFR, while countries in the bottom row are in EMR and SEAR. (For interpretation of the references to colour in this figure legend, the reader is referred to the web version of this article.)

Fig. 2 shows the number of SIAs experienced and the total number of OPV doses reported for children aged 6–59 months in 10 countries representing those that are endemic or recently endemic (Pakistan, India, and Nigeria), with recent outbreaks (Cameroon and Somalia), with high routine immunization (Ghana, Egypt, and Bangladesh), and with low routine immunization (DRC, and Cote D’Ivoire). The top row shows data from countries in the AFR, while the bottom row shows data from countries in the EMR and SEAR. Here, the strength of this relationship varies, but appears generally stronger in those countries in the EMR and SEAR, compared to those in the AFR. Similar figures for all 47 countries are given in the supplementary material.

**Fig. 3:**
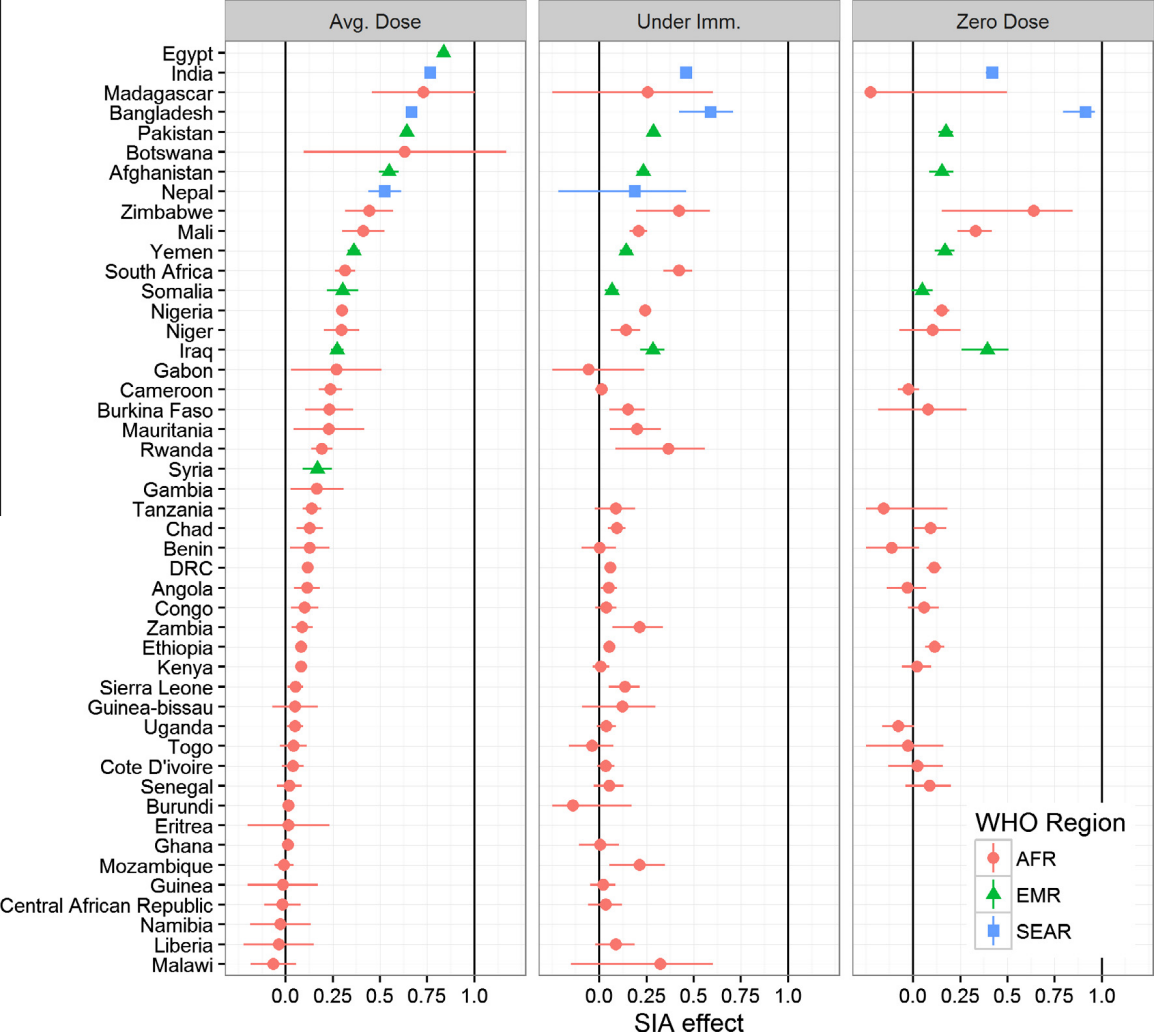
Estimated association between reported number of doses and number of SIAs experienced, based on NP-AFP data between January 2010 and July 2015. From left to right, estimates are from models 1, 2, and 3 detailed in Section 2. Note some countries are excluded from analysis of under-immunized or zero-dose fraction analyses due to insufficient cases.

Fig. 3 summarizes the association between reported doses and SIAs experienced using 3 different models along with 95% confidence intervals for each country analyzed.

When examining the effect of SIAs on the average number of doses reported, we find that 13 of the 47 countries are compatible with the hypothesis that SIAs have no effect on the average number of doses reported. Further, in 30 of the 47 countries we estimate that less than 1 in 4 SIAs experienced will result in a reported dose of vaccine on the case investigation form (i.e. we find the point estimate is less than 0.25). The two remaining countries with wild poliovirus circulation in the EMR, Afghanistan and Pakistan, are among the countries with the strongest response to vaccination campaigns, with 0.55 and 0.65 additional doses reported for each additional campaign, respectively. Nigeria’s estimated response to campaigns is 0.30, which while lower than Afghanistan and Pakistan is higher than the AFR aggregate estimate of 0.15 (Table 2).

We also examined the effect of SIAs on the proportion of children reporting less than 3 doses of OPV (Model 2). This model quantifies the proportion reduction in under-immunized fraction associated with an additional SIA. Again, we find weak associations between reported doses and SIAs, with no discernable effect on the under-immunized fraction in 19 of the 41 countries with sufficient data.

Lastly, we also examined the effect of SIAs on the proportion of children who report zero doses of OPV (Model 3). Here, we find similarly weak associations. In 14 of 26 countries with sufficient data, we found that SIAs had no discernable effect on the zero-dose fraction. In 21 countries, we found that less than 1 in 4 children who report zero doses would report one or more doses after experiencing a campaign. Bangladesh showed the strongest relationship between campaigns and SIAs, where, almost without exception, zero-dose children had not experienced any SIAs.

We estimated the average SIA effect across all countries analyzed within each region, using a simple average of the independent country-level estimates. This analysis is summarized in Table 2. Across all three models, we find that the relationship between SIAs and reported doses is significantly stronger in SEAR than in EMR, and stronger in EMR than in AFR. One explanation for the dramatically attenuated association in AFR is lower campaign coverage. However, monitoring data suggests that polio campaigns vaccinate the vast majority of eligible children in a given area [21]. Therefore, a more plausible explanation is a major difference in the accuracy of reported doses.

### 3.2 Characteristics of vaccination history on case investigation forms

While we do not know how the various survey instruments are implemented by individual surveillance officers, additional information from the case investigation forms give some clues regarding how information is ascertained from a child.

**Fig. 4:**
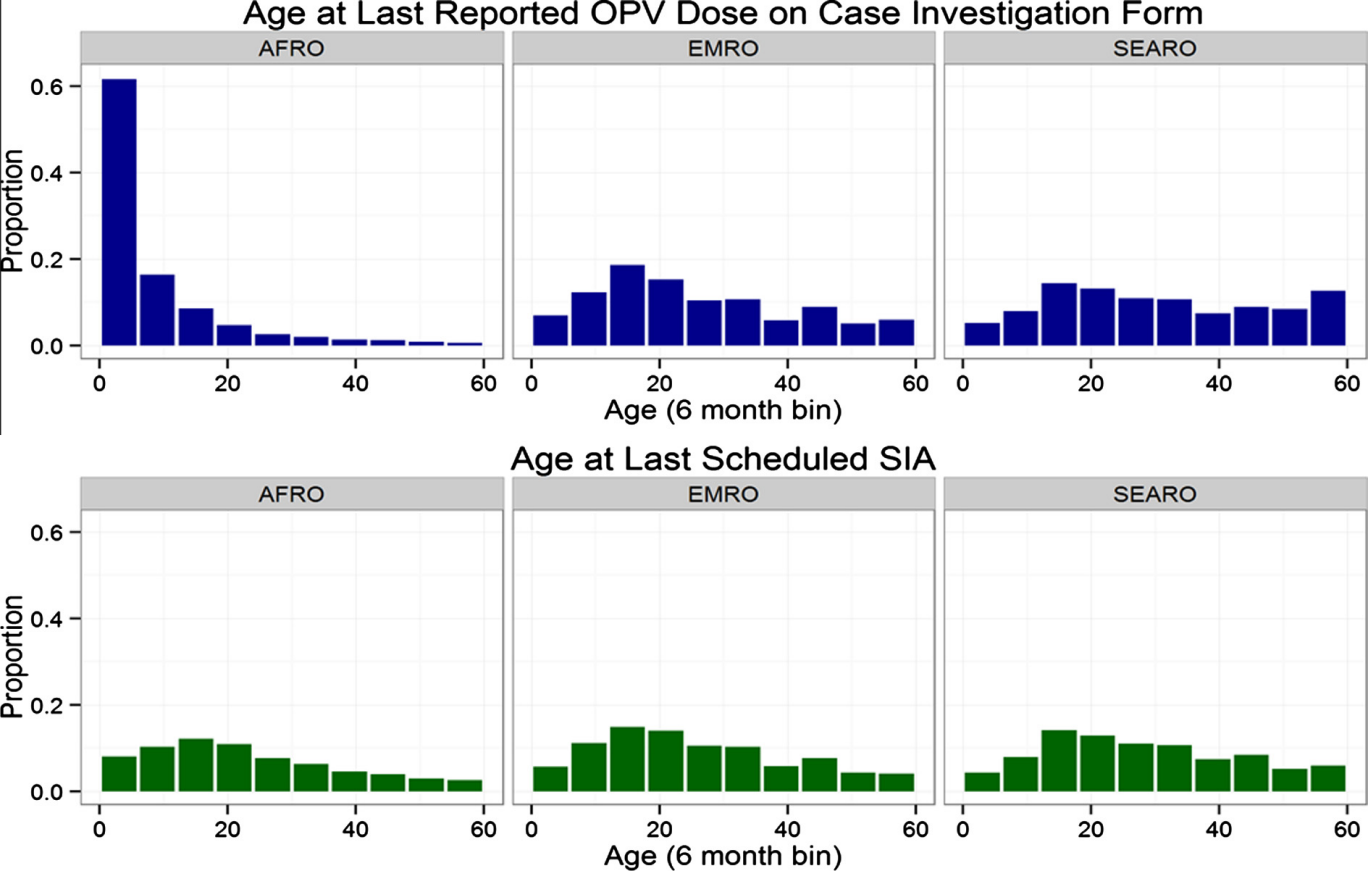
Age of children at last recorded OPV dose (top panel), and last recorded SIA that occurred in their district (bottom panel), from NP-AFP cases with onset between January 2010 and July 2015.

We examined the age at which the most recent OPV dose was received, which is recorded in all regions, though with varying levels of missing data (it is recorded in 31%, 74%, and 67% of AFP cases in AFR, EMR, and SEAR, respectively). We compared this to the child’s age at the last scheduled SIA, and summarized the results in Fig. 4. There, we see that vaccination dates recorded on case-investigation forms in AFR are clustered in the first year of life when routine vaccination is given, whereas in other regions the last vaccination recorded typically comes at an older age. We also found that the median number of SIAs occurring in a child’s district between the last reported OPV dose and date of onset are 4 in AFR, compared with 0 in EMR and SEAR.

This suggests that dates recorded on case investigation forms in AFR often correspond to routine immunization. In contrast, the case investigation forms in EMR and SEAR, which explicitly distinguish between doses delivered through SIAs and routine immunization, report vaccination dates and doses that are more compatible with the number and timing of SIAs.

**Fig. 5:**
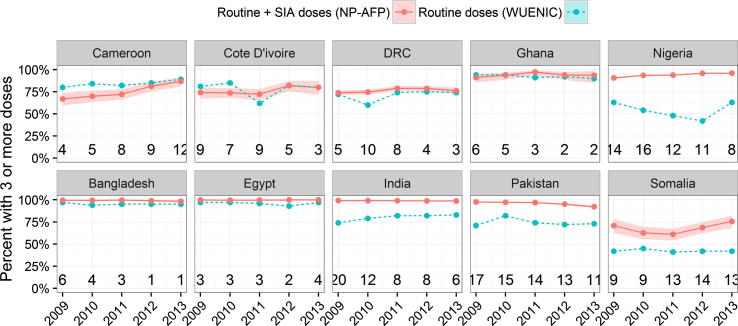
Comparison of fully-immunized fraction from NP-AFP and WUENIC data, by birth cohort. Numbers at bottom of graph indicate median number of SIAs experienced by NP-AFP cases used in the estimate.

In order to investigate this possibility further, we compared the proportion of children reporting 3 or more doses in NP-AFP data, to WUENIC estimates of routine immunization coverage, by birth cohort. Fig. 5 displays this analysis for the 10 countries displayed in Fig. 2, while the remainder of the countries are in the supplemental material. In a number of countries, particularly in AFR, the fraction of NP-AFP cases reporting more than 3 doses of OPV is comparable to or lower than routine immunization coverage, despite many SIAs. Unlike WUENIC estimates, NP-AFP cases are not meant to be representative of the population. Nonetheless, the comparison suggests that doses reported by NP-AFP cases may under-estimate polio immunization levels in some countries. This is most concerning in large countries with low routine immunization levels, such as the DRC, in which SIAs are both epidemiologically critical and also expensive.

## 4 Discussion and conclusion

In this paper, we examined the relationship between SIAs and the number of caregiver-reported polio vaccine doses, which are used to assess polio immunity, risk of spread, and plan vaccination activities. We found that in many countries this association is startlingly weak. One would expect some attenuation of the association due to varying campaign coverage or imperfect recall of vaccination history, but the lack of a discernable relationship between polio vaccine doses and polio vaccination campaigns in many cases prompts some concern about data quality and the proper use of this data in risk assessment.

In current risk models, countries with poor routine immunization coverage and a history of polio importation are often assigned appropriately high risk using available indicators. However, our results show that the level of risk, and the progress made towards reducing that risk through SIAs, is distorted through poor data quality.

Where the association between polio immunization activities childhood immunity indicators is weak, the data suggests that preferential recording of vaccination from routine immunization and under-reporting of vaccinations from SIAs may play a role. To support this claim, we found that in the EMR and SEAR regions of the WHO, where vaccination history is collected through a more extensive questionnaire, reported doses and vaccination campaigns were generally more compatible. Modification of case investigation forms to include more detailed vaccination history has been recommended recently by WHO, which may improve data quality.

Alternative risk assessments and SIA planning processes could be based on supplementing weak routine immunization systems, measured through vaccination records and household surveys, which may be more accurately measured. Further, periodic study of at-risk countries should be done, and the GPEI should consider devoting additional resources for rigorous surveys to improve knowledge of population immunity and SIA effectiveness. Lastly, when planning activities based on any indicator, such as reported vaccine doses, one should validate the indicator by examining its historical relationship to those activities.

## Conflicts of interest

The authors have no conflicts of interest to declare.

## Figures and Tables

**Table 1:** Characteristics of NP-AFP Cases between January 2010 – July 2015 meeting inclusion criteria, by WHO Region.

Region	Countries	NP-AFP cases	Age in months (median, IQR)	% Male	Under-immunized cases (<3 OPV doses) (%)	Zero-dose cases (%)	Total doses (median, IQR)	SIAs experienced (median, IQR)
AFR	37	40,822	18 (12,27)	55	7639 (19%)	1606 (4%)	4 (3,5)	4 (2,7)
EMR	7	16,668	16 (10,24)	57	1593 (10%)	642 (4%)	8 (5,10)	5 (3,8)
SEAR	3	72,335	17 (11,23)	58	3393 (5%)	639 (1%)	8 (6,11)	6 (3,8)

**Table 2:** Summary of association between reported doses and campaigns, based on NP-AFP data between January 2010 and July 2015, by WHO region.

Region	NP-AFP cases	Under-immunized cases	Zero-dose cases	SIA effect on Average Dose (Model 1)		SIA effect on Under-Immunized Fraction (Model 2)		SIA effect on Zero-Dose Fraction (Model 3)	
				Estimate	95% CI	Estimate	95% CI	Estimate	95% CI
AFR	41,179	7790	1650	0.15	(0.13, 0.17)	0.13	(0.10, 0.16)	0.09	(0.02, 0.15)
EMR	16,904	1620	644	0.45	(0.43, 0.47)	0.21	(0.19, 0.22)	0.20	(0.16, 0.23)
SEAR	73,262	3444	647	0.65	(0.62, 0.68)	0.44	(0.33, 0.53)	0.78	(0.65, 0.86)
