# Supplementary material for: Measuring polio immunity to plan immunization activities
Source: Vaccine. 2016 Nov 21;34(48):5946–52. doi: 10.1016/j.vaccine.2016.10.017 (PMC5104692; doi:10.1016/j.vaccine.2016.10.017)
Supplement: Supplementary data 1 [file mmc1.docx]

# Supplementary material

## Survey Instruments

Below are example survey instruments used in the three WHO regions studied. The most extensive and specific questionnaire is used in Pakistan, whereas the instrument used in the African region is the least extensive. Notably, the instrument used in the African region does not distinguish between SIA and Routine doses, and leaves much of the elicitation process to the surveillance officer’s discretion.

Figure S1: Example survey instruments used to assess polio vaccination history. Top panel: survey instrument used in Pakistan. Middle panel: survey instrument used in AFR. Bottom panel: survey instrument used in India


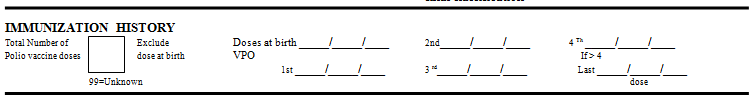


## Description of the data

Tables below summarize the NP-AFP data in the 47 countries examined, before applying exclusion criteria. We see that the typical number of SIAs experienced by NP-AFP cases varies widely by country with the intensity of SIAs carried out in those countries (median of 28 in India, compared to a median of 0 in Madagascar). There is notably less variability in the total number of OPV doses reported. In AFR, the median number of doses is typically 3 or 4, even in countries such as Chad or DRC where the median number of SIAs experienced is large. On the other hand, in EMR and SEAR, the median number of doses reported is large in countries where many campaigns have occurred (e.g. in Afghanistan and India).

The discrepancy between reported doses and SIAs experienced in countries with many campaigns could be explained by recall error. After experiencing 28 campaigns (the median in India), it is understandably difficult to recall how many doses have been received without precise records, which may explain why the median reported doses are 15. It is for this reason that we limited the analysis to those children who had experienced fewer than 10 SIAs.

| **Region** | **Countries** | **NP-AFP Cases** | **Age in months (median, IQR)** | **% Male** | **Under-immunized (%)** | **Zero-dose Cases (%)** | **Total Doses (median, IQR)** | **SIAs Experienced (median, IQR)** |
| --- | --- | --- | --- | --- | --- | --- | --- | --- |
| AFR | 37 | 92,754 | 31 (18,58) | 56 | 13,753 (15%) | 3928 (4%) | 4 (3,7) | 9 (3,18) |
| EMR | 7 | 50,232 | 36 (18,60) | 58 | 3063 (6%) | 1498 (3%) | 10 (7,15) | 13 (6,25) |
| SEAR | 3 | 314,206 | 46 (23,83) | 58 | 6490 (2%) | 2606 (1%) | 15 (10,24) | 27 (11,42) |

Table S1: Summary of NP-AFP Cases before applying exclusion criteria on SIAs, Age, and reported Doses.

|  |  | **NP-AFP Cases** | | | |  | **Age** | | **SIAs** | | **Total Doses** | |
| --- | --- | --- | --- | --- | --- | --- | --- | --- | --- | --- | --- | --- |
| **Region** | **Country** | **Total** | **Meeting Exclusion criteria** | **Total Under Immunized** | **Total Zero Dose** | **% Male** | **median** | **IQR** | **median** | **IQR** | **Median** | **IQR** |
| AFR | Angola | 1693 | 671 | 200 | 57 | 55 | 32 | (19, 62) | 11 | (4, 19) | 3 | (2, 6) |
| AFR | Benin | 635 | 395 | 86 | 44 | 51 | 32 | (20, 48) | 8 | (6, 12) | 4 | (3, 6) |
| AFR | Botswana | 94 | 53 | 4 | 1 | 55 | 41 | (24, 74) | 0 | (0, 1) | 4 | (3, 4) |
| AFR | Burkina Faso | 1429 | 518 | 44 | 10 | 59 | 36 | (24, 60) | 12 | (8, 17) | 5 | (4, 8) |
| AFR | Burundi | 415 | 314 | 12 | 0 | 55 | 31 | (20, 56) | 2 | (0, 4) | 3 | (3, 3) |
| AFR | Cameroon | 2333 | 881 | 240 | 113 | 55 | 48 | (25, 108) | 7 | (3, 10) | 3 | (1, 4) |
| AFR | CAR | 420 | 159 | 41 | 4 | 56 | 36 | (19, 67) | 11 | (6, 15) | 3 | (3, 4) |
| AFR | chad | 2283 | 470 | 108 | 43 | 55 | 36 | (22, 60) | 16 | (10, 22) | 4 | (3, 6) |
| AFR | Congo | 625 | 235 | 80 | 45 | 57 | 61 | (30, 144) | 6 | (1, 10) | 3 | (0, 4) |
| AFR | cote d'ivoire | 1679 | 721 | 187 | 29 | 55 | 28 | (18, 52) | 8 | (4, 13) | 3 | (2, 4) |
| AFR | DRC | 9109 | 4556 | 1310 | 328 | 55 | 35 | (20, 62) | 8 | (3, 12) | 3 | (2, 5) |
| AFR | Eritrea | 324 | 63 | 6 | 1 | 59 | 48 | (25, 97) | 0 | (0, 0) | 3 | (3, 3) |
| AFR | Ethiopia | 4723 | 2314 | 746 | 203 | 57 | 48 | (24, 86) | 6 | (3, 11) | 3 | (2, 4) |
| AFR | Gabon | 122 | 59 | 15 | 8 | 59 | 36 | (18, 86) | 3 | (1, 3) | 3 | (2, 4) |
| AFR | Gambia | 184 | 96 | 6 | 2 | 68 | 36 | (24, 66) | 4 | (0, 7) | 5 | (4, 5) |
| AFR | Ghana | 1295 | 783 | 50 | 0 | 59 | 30 | (19, 52) | 6 | (3, 10) | 4 | (3, 4) |
| AFR | Guinea | 969 | 345 | 72 | 1 | 59 | 36 | (22, 59) | 10 | (7, 16) | 4 | (3, 5) |
| AFR | Guinea-Bissau | 64 | 51 | 12 | 1 | 50 | 24 | (16, 38) | 3 | (2, 5) | 3 | (3, 4) |
| AFR | Kenya | 2926 | 1290 | 154 | 43 | 52 | 48 | (24, 88) | 3 | (0, 8) | 3 | (3, 4) |
| AFR | Liberia | 222 | 98 | 32 | 1 | 54 | 30 | (19, 58) | 8 | (5, 13) | 3 | (2, 4) |
| AFR | Madagascar | 1404 | 929 | 142 | 48 | 54 | 29 | (16, 59) | 0 | (0, 0) | 3 | (3, 3) |
| AFR | Malawi | 409 | 222 | 15 | 0 | 55 | 49 | (26, 100) | 0 | (0, 2) | 3 | (3, 3) |
| AFR | Mali | 1150 | 371 | 132 | 87 | 61 | 36 | (19, 60) | 12 | (7, 18) | 4 | (3, 6) |
| AFR | Mauritania | 298 | 160 | 19 | 3 | 51 | 36 | (21, 60) | 8 | (5, 11) | 4 | (3, 5) |
| AFR | Mozambique | 961 | 704 | 90 | 6 | 52 | 25 | (16, 44) | 2 | (0, 2) | 3 | (3, 3) |
| AFR | Namibia | 113 | 57 | 6 | 1 | 53 | 40 | (23, 131) | 4 | (2, 7) | 4 | (3, 5) |
| AFR | Niger | 1719 | 663 | 67 | 16 | 55 | 27 | (19, 42) | 12 | (8, 18) | 5 | (4, 9) |
| AFR | Nigeria | 48,202 | 16,554 | 2613 | 279 | 57 | 27 | (17, 48) | 15 | (7, 25) | 6 | (4, 10) |
| AFR | Rwanda | 875 | 443 | 20 | 5 | 57 | 54 | (27, 108) | 2 | (1, 4) | 3 | (3, 4) |
| AFR | Senegal | 757 | 498 | 76 | 35 | 57 | 30 | (18, 55) | 4 | (2, 6) | 3 | (3, 4) |
| AFR | Sierra Leone | 748 | 348 | 37 | 2 | 49 | 30 | (17, 48) | 9 | (5, 14) | 3 | (3, 4) |
| AFR | South Africa | 1483 | 989 | 277 | 0 | 52 | 33 | (19, 61) | 2 | (0, 2) | 3 | (3, 4) |
| AFR | Togo | 469 | 311 | 40 | 10 | 52 | 36 | (22, 64) | 6 | (3, 8) | 4 | (3, 4) |
| AFR | Uganda | 2754 | 1752 | 409 | 172 | 57 | 34 | (19, 70) | 2 | (1, 3) | 3 | (3, 4) |
| AFR | Tanzania | 2964 | 1826 | 216 | 30 | 54 | 36 | (20, 66) | 0 | (0, 2) | 3 | (3, 4) |
| AFR | Zambia | 694 | 456 | 72 | 3 | 52 | 39 | (23, 70) | 1 | (0, 3) | 3 | (3, 4) |
| AFR | Zimbabwe | 742 | 467 | 79 | 14 | 52 | 34 | (18, 73) | 1 | (0, 1) | 4 | (3, 4) |
| EMR | Afghanistan | 10816 | 4085 | 342 | 96 | 56 | 36 | (18, 70) | 10 | (5, 19) | 12 | (8, 18) |
| EMR | Egypt | 5328 | 3769 | 7 | 0 | 57 | 28 | (18, 55) | 4 | (2, 7) | 10 | (8, 13) |
| EMR | Iraq | 2509 | 1171 | 105 | 30 | 60 | 31 | (18, 60) | 10 | (5, 14) | 6 | (4, 8) |
| EMR | Pakistan | 28425 | 5473 | 624 | 254 | 59 | 34 | (18, 60) | 20 | (11, 31) | 11 | (9, 15) |
| EMR | Somalia | 1616 | 506 | 201 | 120 | 59 | 25 | (15, 48) | 13 | (7, 20) | 4 | (1, 8) |
| EMR | Syria | 1009 | 165 | 9 | 1 | 58 | 48 | (22, 87) | 7 | (2, 10) | 5 | (5, 6) |
| EMR | Yemen | 2691 | 1499 | 321 | 141 | 59 | 36 | (18, 75) | 6 | (4, 10) | 5 | (3, 8) |
| SEAR | Bangladesh | 8658 | 4346 | 95 | 23 | 59 | 48 | (25, 93) | 7 | (3, 15) | 10 | (7, 14) |
| SEAR | India | 307171 | 67347 | 3284 | 616 | 58 | 46 | (23, 83) | 28 | (12, 42) | 15 | (10, 24) |
| SEAR | Nepal | 3093 | 642 | 14 | 2 | 61 | 58 | (26, 107) | 0 | (0, 8) | 9 | (6, 11) |

Table S2: Detailed summary of Country data, before applying exclusion criteria on SIAs, Age, and reported Doses

## Detailed Country-Level Results

Table S3 below gives the detailed results for the 3 models, which is graphically summarized in Figure 2 of the manuscript. In addition, Figure S2 shows scatterplots of the relationship between SIAs.

| **Region** | **Country** | **NP-AFP Cases** | **Under-Immunized Cases** | **Zero-Dose Cases** | **Model 1:**  **SIA Effect on average doses**  **Coefficient (95% CI)** | **Model 2:**  **SIA effect on under-immunized fraction**  **Coefficient (95% CI)** | **Model 3:**  **SIA effect on zero-dose fraction**  **Coefficient (95% CI)** |
| --- | --- | --- | --- | --- | --- | --- | --- |
| AFR | Angola | 671 | 200 | 57 | 0.11 (0.05, 0.18) | 0.05 (0.01, 0.09) | -0.03 (-0.14, 0.07) |
| AFR | Benin | 395 | 86 | 44 | 0.13 (0.02, 0.23) | 0.00 (-0.09, 0.09) | -0.11 (-0.28, 0.03) |
| AFR | Botswana | 53 | 4 | 1 | 0.63 (0.09, 1.17) | NA | NA |
| AFR | Burkina Faso | 518 | 44 | 10 | 0.23 (0.10, 0.36) | 0.15 (0.05, 0.24) | 0.08 (-0.18, 0.28) |
| AFR | Burundi | 314 | 12 | 0 | 0.02 (-0.01, 0.04) | -0.14 (-0.57, 0.17) | NA |
| AFR | Cameroon | 881 | 240 | 113 | 0.24 (0.18, 0.30) | 0.01 (-0.02, 0.05) | -0.02 (-0.08, 0.03) |
| AFR | CAR | 159 | 41 | 4 | -0.02 (-0.11, 0.08) | 0.03 (-0.06, 0.12) | NA |
| AFR | Chad | 470 | 108 | 43 | 0.13 (0.06, 0.20) | 0.09 (0.04, 0.14) | 0.09 (0.00, 0.18) |
| AFR | Congo | 235 | 80 | 45 | 0.10 (0.03, 0.17) | 0.04 (-0.02, 0.09) | 0.06 (-0.03, 0.14) |
| AFR | Cote D'ivoire | 721 | 187 | 29 | 0.04 (-0.02, 0.10) | 0.04 (-0.01, 0.08) | 0.02 (-0.13, 0.16) |
| AFR | DRC | 4556 | 1310 | 328 | 0.12 (0.10, 0.14) | 0.06 (0.04, 0.07) | 0.11 (0.07, 0.15) |
| AFR | Eritrea | 63 | 6 | 1 | 0.02 (-0.20, 0.23) | NA | NA |
| AFR | Ethiopia | 2314 | 746 | 203 | 0.08 (0.06, 0.11) | 0.05 (0.03, 0.07) | 0.12 (0.06, 0.17) |
| AFR | Gabon | 59 | 15 | 8 | 0.27 (0.03, 0.51) | -0.06 (-0.47, 0.24) | NA |
| AFR | Gambia | 96 | 6 | 2 | 0.17 (0.02, 0.31) | NA | NA |
| AFR | Ghana | 783 | 50 | 0 | 0.01 (-0.01, 0.04) | 0.00 (-0.11, 0.10) | NA |
| AFR | Guinea | 345 | 72 | 1 | -0.02 (-0.20, 0.17) | 0.02 (-0.05, 0.09) | NA |
| AFR | Guinea-Bissau | 51 | 12 | 1 | 0.05 (-0.07, 0.17) | 0.12 (-0.09, 0.30) | NA |
| AFR | Kenya | 1290 | 154 | 43 | 0.08 (0.05, 0.11) | 0.01 (-0.04, 0.05) | 0.02 (-0.06, 0.10) |
| AFR | Liberia | 98 | 32 | 1 | -0.04 (-0.22, 0.15) | 0.09 (-0.02, 0.19) | NA |
| AFR | Madagascar | 929 | 142 | 48 | 0.73 (0.46, 1.00) | 0.26 (-0.39, 0.60) | -0.23 (-2.00, 0.50) |
| AFR | Malawi | 222 | 15 | 0 | -0.06 (-0.18, 0.05) | 0.32 (-0.15, 0.60) | NA |
| AFR | Mali | 371 | 132 | 87 | 0.41 (0.30, 0.52) | 0.21 (0.16, 0.25) | 0.33 (0.23, 0.42) |
| AFR | Mauritania | 160 | 19 | 3 | 0.23 (0.04, 0.42) | 0.20 (0.06, 0.32) | NA |
| AFR | Mozambique | 704 | 90 | 6 | -0.01 (-0.06, 0.04) | 0.21 (0.05, 0.35) | NA |
| AFR | Namibia | 57 | 6 | 1 | -0.03 (-0.19, 0.13) | NA | NA |
| AFR | Niger | 663 | 67 | 16 | 0.30 (0.20, 0.39) | 0.14 (0.06, 0.22) | 0.10 (-0.07, 0.25) |
| AFR | Nigeria | 16554 | 2613 | 279 | 0.30 (0.29, 0.31) | 0.24 (0.23, 0.25) | 0.15 (0.11, 0.19) |
| AFR | Rwanda | 443 | 20 | 5 | 0.19 (0.14, 0.25) | 0.37 (0.09, 0.56) | NA |
| AFR | Senegal | 498 | 76 | 35 | 0.02 (-0.05, 0.09) | 0.05 (-0.03, 0.13) | 0.09 (-0.04, 0.20) |
| AFR | Sierra Leone | 348 | 37 | 2 | 0.05 (0.01, 0.09) | 0.14 (0.05, 0.21) | NA |
| AFR | South Africa | 989 | 277 | 0 | 0.32 (0.26, 0.37) | 0.42 (0.34, 0.49) | NA |
| AFR | Togo | 311 | 40 | 10 | 0.04 (-0.03, 0.11) | -0.04 (-0.16, 0.07) | -0.03 (-0.26, 0.16) |
| AFR | Uganda | 1752 | 409 | 172 | 0.05 (0.01, 0.09) | 0.04 (-0.01, 0.09) | -0.08 (-0.16, 0.00) |
| AFR | Tanzania | 1826 | 216 | 30 | 0.14 (0.09, 0.19) | 0.09 (-0.02, 0.19) | -0.16 (-0.64, 0.18) |
| AFR | Zambia | 456 | 72 | 3 | 0.09 (0.03, 0.14) | 0.21 (0.07, 0.34) | NA |
| AFR | Zimbabwe | 467 | 79 | 14 | 0.44 (0.32, 0.57) | 0.42 (0.20, 0.59) | 0.64 (0.15, 0.85) |
| EMR | Afghanistan | 4085 | 342 | 96 | 0.55 (0.49, 0.60) | 0.23 (0.20, 0.27) | 0.15 (0.09, 0.21) |
| EMR | Egypt | 3769 | 7 | 0 | 0.84 (0.81, 0.86) | NA | NA |
| EMR | Iraq | 1171 | 105 | 30 | 0.27 (0.24, 0.31) | 0.28 (0.22, 0.34) | 0.39 (0.26, 0.51) |
| EMR | Pakistan | 5473 | 624 | 254 | 0.64 (0.62, 0.67) | 0.29 (0.26, 0.31) | 0.17 (0.13, 0.21) |
| EMR | Somalia | 506 | 201 | 120 | 0.30 (0.22, 0.39) | 0.07 (0.03, 0.10) | 0.05 (-0.01, 0.10) |
| EMR | Syria | 165 | 9 | 1 | 0.17 (0.09, 0.25) | NA | NA |
| EMR | Yemen | 1499 | 321 | 141 | 0.36 (0.33, 0.39) | 0.14 (0.11, 0.17) | 0.17 (0.12, 0.22) |
| SEAR | Bangladesh | 4346 | 95 | 23 | 0.67 (0.65, 0.69) | 0.59 (0.42, 0.71) | 0.91 (0.79, 0.96) |
| SEAR | India | 67347 | 3284 | 616 | 0.77 (0.76, 0.78) | 0.46 (0.45, 0.47) | 0.42 (0.39, 0.45) |
| SEAR | Nepal | 642 | 14 | 2 | 0.53 (0.44, 0.61) | 0.19 (-0.22, 0.46) | NA |

Table S3: Detailed country-level results for Models 1, 2, and 3.

##
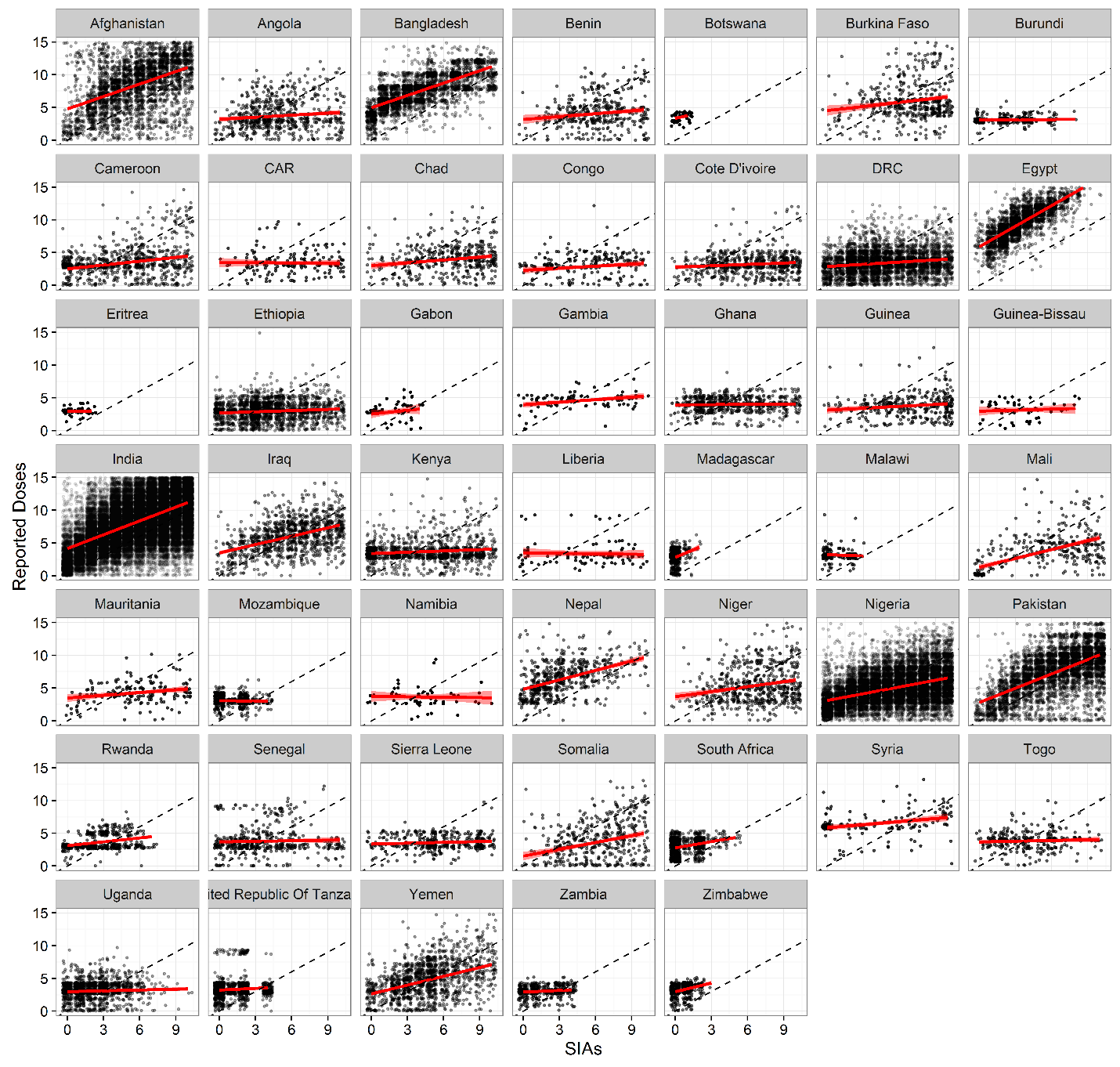


Figure S2: Total number of OPV doses reported and the number of vaccination campaigns experienced by a child, based on NP-AFP data between January 2010 – July 2015. Red lines indicate simple linear regression fit and 95% confidence interval.

## Relationship between reported polio doses and routine immunization levels


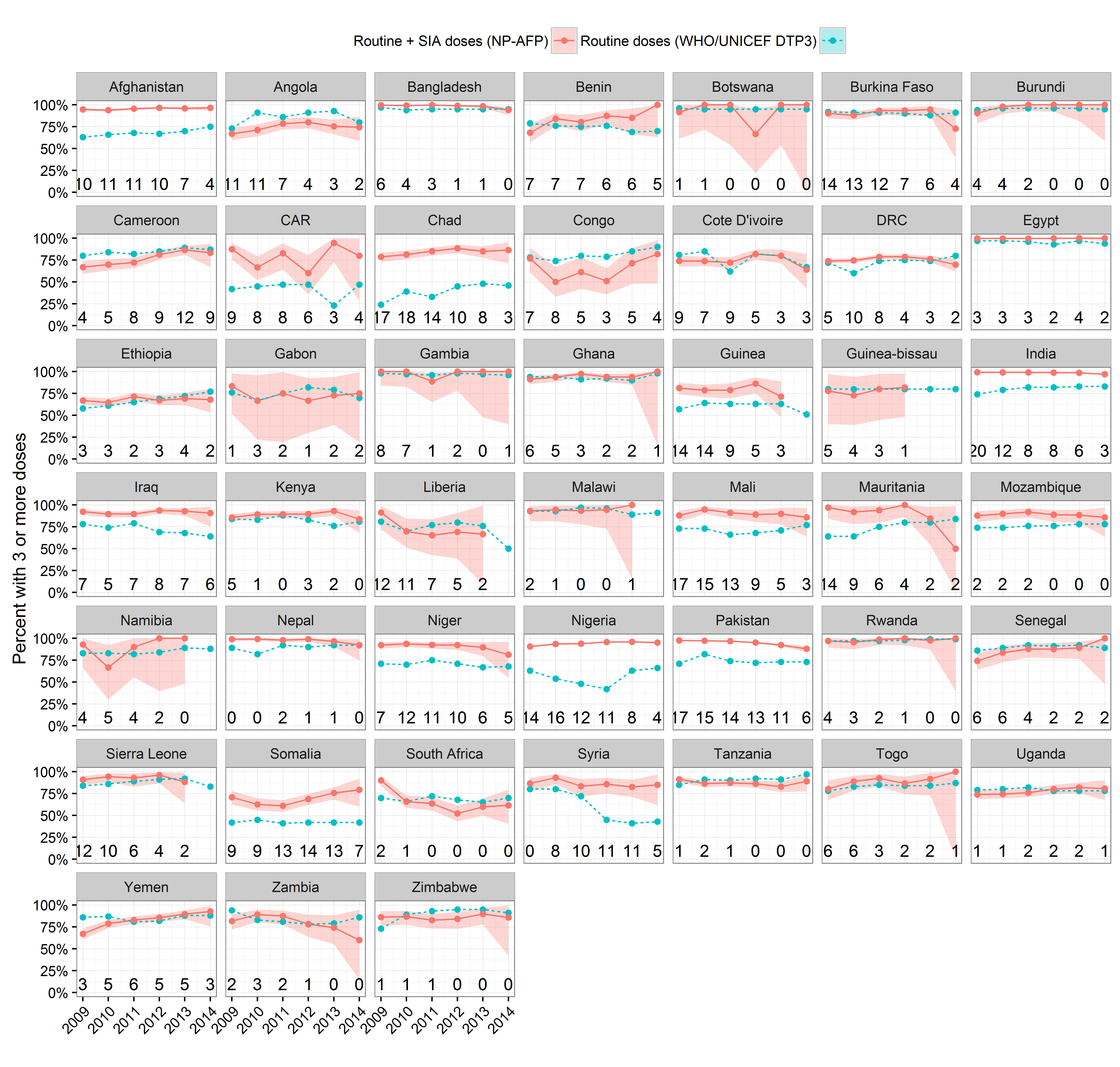
Here, we examine the relationship between routine immunization coverage and reported doses among NP-AFP cases as an informal measure of the impact of SIAs. We used the WHO/UNICEF best estimates of national DTP3 coverage to estimate the proportion of the population that receive 3 or more doses of polio in the absence of SIAs. We compared these routine coverage estimates for a given year to the fraction of NP-AFP cases born in that year who report 3 or more doses of OPV, and restricted our analysis to NP-AFP cases who were older than 6 months at the time dose history was ascertained. While these estimates are not precisely comparable - neither are from population-based surveys, and vaccination history is not obtained using the same questionnaire - some interesting patterns are apparent.

Figure S3: Comparison between total reported doses (from NP-AFP data) and WHO/UNICEF estimates of routine immunization coverage, by birth year. Numbers at the bottom of the plot give the number of SIAs conducted that year.

In a number of countries (e.g. Afghanistan, Chad, India, Iraq, Nigeria, Niger, Pakistan, and Somalia) the fraction of NP-AFP cases reporting 3 or more OPV doses is substantially higher than estimated routine immunization levels, suggesting impact of SIAs. In most of these countries (e.g. Afghanistan, India, Iraq, Nigeria, and Pakistan) we also estimate relatively strong effects of SIAs on the under-immunized fraction. In others (e.g. Chad, Somalia) we estimate small effects of SIAs on under-immunized fraction. In Somalia, this discrepancy is likely due to inaccessibility: areas of southern Somalia are under-immunized despite many scheduled campaigns, leading to a weak overall association.

In Chad, where the impact of SIAs on both average doses and the under-immunized fraction is low, additional issues seem to be at play. There, it seems the first few SIAs a child experienced will influence the number of doses reported: among children who have experienced 3 or fewer SIAs, only 53% report 3 or more doses of OPV, while among those who have experienced 4 or more SIAs 81% report 3 or more doses. However, the average number of doses is roughly constant for children who have experienced 4 or more SIAs. This may be an artifact of poor recall and the survey instrument used, as it is unlikely that vaccine would be withheld from children who have experienced 4 or more campaigns. Indeed, examining the survey instrument used in AFRO, the dates of only the first 4 doses of OPV are elicited, which may lead to attenuation of reported doses for those experiencing many SIAs and a corresponding attenuated association with SIAs.

In many other countries the under-immunized fraction from NP-AFP data is comparable to or lower than estimated routine immunization coverage, despite the fact that NP-AFP data should include both routine and SIA doses. Notably, this is true in 4 of the 5 most populous nations in AFR (Ethiopia, the Democratic Republic of the Congo, Tanzania, and South Africa), where SIAs are both expensive and critical. For some of these countries (e.g. Tanzania and South Africa) few polio SIAs are conducted, and thus one would not expect a large difference between routine coverage and the fraction of children reporting 3 or more doses when including SIAs. For other countries where many SIAs have been conducted (e.g. the Democratic Republic of the Congo and Ethiopia), the concordance suggests the possibility that survey-instruments may not record doses received through SIAs, or that WHO/UNICEF estimates of coverage are biased upwards.

## Sensitivity Analyses

In this section we examine the sensitivity of our primary analysis to the removal of children who were eligible for more than 10 SIAs. This exclusion was applied since we do not expect children who have experience many SIAs to be able to reliably recall the number of SIAs in which they received vaccine, regardless of how dose history is elicited. This lack of accuracy among children who experienced many campaigns may attenuate the association between reported doses and SIAs, and have a larger effect in countries implementing many SIAs.

Removing this restriction resulted in 298,950 children in the analysis, compared to 129,825 cases in the original analysis. Of the cases removed by the restriction, 72% were from India, 13% from Pakistan, and 8% from Nigeria.


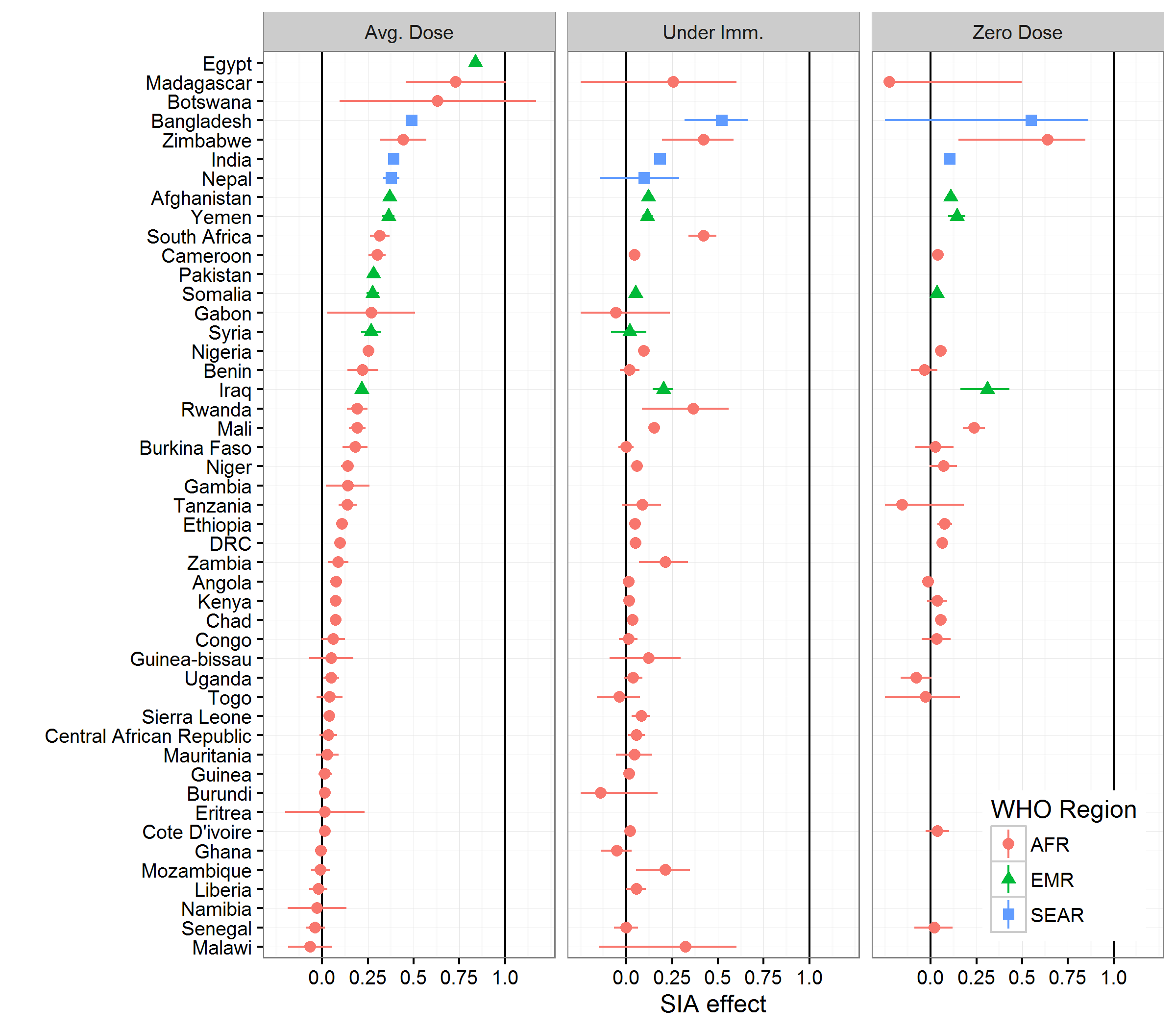


Figure S4: Estimated association between reported number of doses and number of SIAs experienced, based on NP-AFP data between January 2010 and July 2015, including those eligible for 10 or more SIAs. From left to right, estimates are from Models 1, 2, and 3 detailed in Section 2.

Figure S5 shows the effect estimates from Model 1, 2, and 3 applied to this larger dataset, analogous to Figure 3 in the manuscript. Since more cases are available for analysis, confidence intervals are narrower. Trends in the association between reported doses and SIAs are qualitatively similar in this sensitivity analysis, where many countries, particularly in AFR, demonstrate weak relationships. However, the association between reported doses and SIAs is notably weaker in India, Bangladesh, and Pakistan across all models. For instance, in India the increase in the number of doses per SIA changed from 0.77 to 0.39 in the sensitivity analysis. There, 25% of children in the expanded dataset had experienced 29 or more SIAs. One might expect under-reporting of doses among these children, regardless of how they are elicited, resulting in this attenuated relationship. Figure S4 summarizes the difference in effect estimates in the sensitivity analysis. Points below the diagonal line indicate attenuation of the association, which is most pronounced for India, Bangladesh, and Pakistan, countries that implemented many SIAs in the years considered.


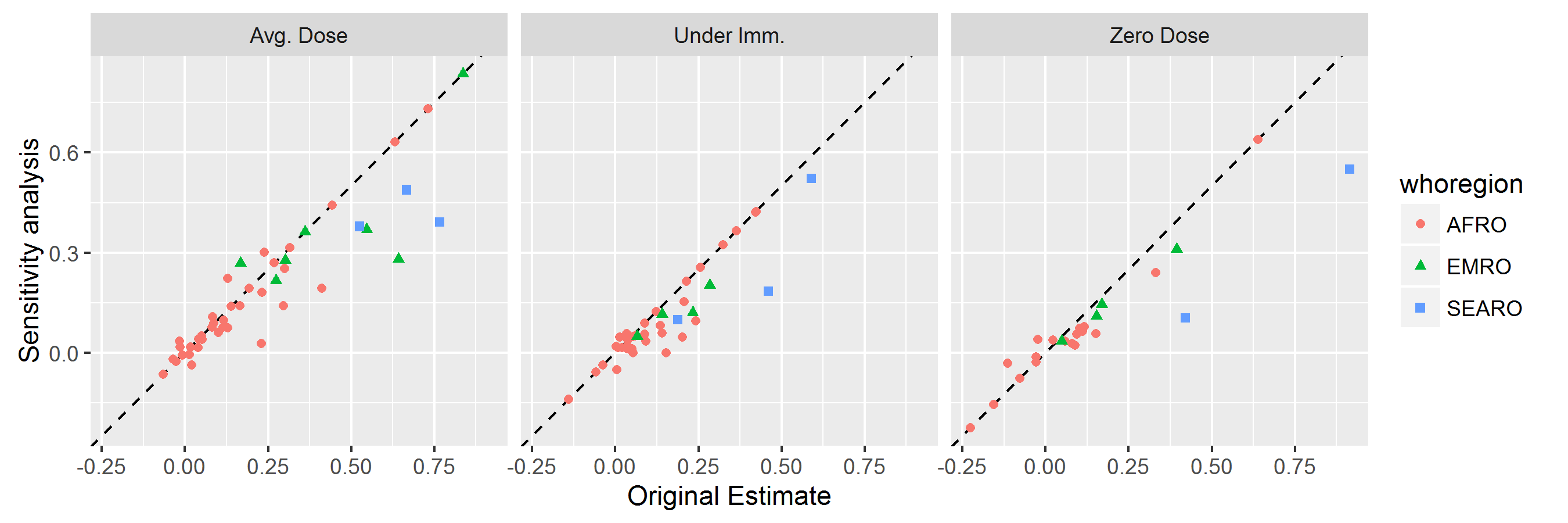


Figure S5: Comparison between effect estimates from the primary analysis (X-axis) which excludes NP-AFP cases with > 10 SIAs, and a sensitivity analysis which does not exclude those cases (Y-axis) .

## Country-level predictors of campaign effectiveness

A reviewer helpfully suggested examining other covariates that might explanation country-level differences in campaign effectiveness, such as coverage through routine immunization or how recently the country was considered endemic.

The goal of this analysis is not to exhaustively examine all predictors and transformations thereof, but to check the plausibility of a selection of relevant predictors through simple means.

In addition to WHO region, we compiled 6 predictors and ran simple regressions of the estimated effectiveness against each. We include only estimated effectiveness on average doses. We summarize these in Table S4 and Figure S6. Table S4 summarizes the relationship of the predictors with estimated effectiveness with $R^{2}$ (the between-country variation in effectiveness explained by the given predictor), Pearson correlation ($\sqrt{R^{2}}$ for univariate regression), and a 95% confidence interval on correlation. We have used correlation instead of regression coefficients in order to give a scale-independent measure of the linear relationship.

| **Predictor** | $\boldsymbol{R}^{\boldsymbol{2}}$ | **Correlation** | **Correlation 95% CI** |
| --- | --- | --- | --- |
| GPEI surveillance 2016 (US, millions) | 0.08 | 0.29 | (0.00, 0.53) |
| GPEI surveillance 2016 (US, millions per 1000 children < 5) | 0.00 | 0.06 | (-0.23, 0.34) |
| Total Health Expenditure (THE) per Capita in US$ | 0.01 | 0.12 | (-0.18, 0.39) |
| Total Health Expenditure (THE) per Capita in Int$ (Purchasing Power Parity) | 0.06 | 0.24 | (-0.05, 0.50) |
| NID equivalent campaigns 2010-2015 | 0.02 | 0.14 | (-0.15, 0.41) |
| DTP3 2010-2105 | 0.01 | 0.07 | (-0.22, 0.35) |
| WHO Region (AFR, EMR, SEAR) | 0.42 | - | - |

Table S4: Summary of the relationship between predictors and estimated effectiveness (number of doses).

The regression on WHO region finds an $R^{2}$ of 0.42, albeit with an extra parameter (three levels of the factor, compared to two parameters for the intercept and slope for the other predictors). This far exceeds the model fit of the other predictors, where GPEI surveillance budget yields the second strongest association with an $R^{2}$ of 0.08. With the exception of WHO region, the statistical evidence for an association is questionable.

We provide a brief description and rationale for the predictors.

- “GPEI surveillance 2016…” refers to the stated surveillance budget by country in GPEI documents, available at <http://www.polioeradication.org/Portals/0/Document/Financing/D.pdf>. The rationale for these predictors is that greater expenditure on surveillance is an indicator of either more programmatic attention, which could result in better campaigns and/or less biased reporting of doses. We did not have ready access to a more relevant time period (2010-2015), so took 2016 as a proxy for programmatic attention that may have existed earlier. We further branch to two predictors:
  - Budget in absolute terms. This tends to be directed more towards more populous countries (e.g. Ethiopia) and/or recently endemic countries (e.g. Afghanistan, Nigeria). Oddly this is arguably the best of the considered predictors.
  - Budget in relative terms, i.e. per 1000 children. This predictor demonstrated very little relationship with the outcome.
- “Total Health Expenditure…” refers to measures of per capita health expenditure, averaged from 2010 to 2015. This is available from the Global Health Expenditure Database from the WHO (<http://apps.who.int/nha/database>). The rationale for this is twofold: first that the robustness of the health system ecosystem could be related to campaign efficacy and/or that a well-funded “front line” network of primary health care providers and the general health system ecosystem will be better trained to solicit dose reporting. This is expressed in two metrics, first in US$ and second in purchasing power parity terms. Neither explains a compelling amount of variation in campaign effectiveness.
- “NID equivalent campaigns…” counts the number of vaccination campaigns from 2010-2015 in terms of the proportion of population covered, for example such that a national campaign will be 1 and a subnational campaign will be counted as the fraction of population targeted. The data comes from the WHO. The rationale is that more frequent campaigns may result in a more effective campaigns (infrastructure, expertise) and/or a greater awareness of campaigns by surveillance personnel resulting in less bias in reporting. This has very little relationship with the predictor.
- “DTP3…” comes from WHO/UNICEF estimates, averaged from 2010-2015. One might wonder if high DTP3 is indicative of a well-functioning health care system or health seeking behavior which might result in well-functioning or high-participation campaigns. Alternatively, high DTP3 might suggest increasing apathy with respect to the need for campaigns. Either way, it has very little evidence of a relationship with effectiveness.

Many of these indicators may be related with endemic or recent endemic status, for example low DTP3 suggesting low routine immunization and fertile outbreak conditions or the number of SIA indicative of endemic status or outbreak risk. However, none result in compelling relationships, either numerically by explained variance or by extension, graphically (see Figure S6).

Figure S6: Campaign effectiveness estimates regressed against other country features.
